# Genomics and synthetic community experiments uncover the key metabolic roles of acetic acid bacteria in sourdough starter microbiomes

**DOI:** 10.1128/msystems.00537-24

**Published:** 2024-09-17

**Authors:** H. B. Rappaport, Nimshika P. J. Senewiratne, Sarah K. Lucas, Benjamin E. Wolfe, Angela M. Oliverio

**Affiliations:** 1 Department of Biology, Syracuse University, Syracuse, New York, USA; 2 Department of Biology, Tufts University, Medford, Massachusetts, USA; Pontificia Universidad Catolica de, Santiago, Santiago, Chile

**Keywords:** acetic acid bacteria, sourdough starter, synthetic communities, comparative genomics, strain diversity, microbiome

## Abstract

While research on the sourdough microbiome has primarily focused on lactic acid bacteria (LAB) and yeast, recent studies have found that acetic acid bacteria (AAB) are also common members. However, the ecology, genomic diversity, and functional contributions of AAB in sourdough remain unknown. To address this gap, we sequenced 29 AAB genomes, including three that represent putatively novel species, from a collection of over 500 sourdough starters surveyed globally from community scientists. We found variations in metabolic traits related to carbohydrate utilization, nitrogen metabolism, and alcohol production, as well as in genes related to mobile elements and defense mechanisms. Sourdough AAB genomes did not cluster when compared to AAB isolated from other environments, although a subset of gene functions was enriched in sourdough isolates. The lack of a sourdough-specific genomic cluster may reflect the nomadic lifestyle of AAB. To assess the consequences of AAB on the emergent function of sourdough starter microbiomes, we constructed synthetic starter microbiomes, varying only the AAB strain included. All AAB strains increased the acidification of synthetic sourdough starters relative to yeast and LAB by 18.5% on average. Different strains of AAB had distinct effects on the profile of synthetic starter volatiles. Taken together, our results begin to define the ways in which AAB shape emergent properties of sourdough and suggest that differences in gene content resulting from intraspecies diversification can have community-wide consequences on emergent function.

**IMPORTANCE:**

This study is a comprehensive genomic and ecological survey of acetic acid bacteria (AAB) isolated from sourdough starters. By combining comparative genomics with manipulative experiments using synthetic microbiomes, we demonstrate that even strains with >97% average nucleotide identity can shift important microbiome functions, underscoring the importance of species and strain diversity in microbial systems. We also demonstrate the utility of sourdough starters as a model system to understand the consequences of genomic diversity at the strain and species level on multispecies communities. These results are also relevant to industrial and home-bakers as we uncover the importance of AAB in shaping properties of sourdough starters that have direct impacts on sensory notes and the quality of sourdough bread.

## INTRODUCTION

Acetic acid bacteria (AAB) are an important member of many fermented food microbiomes. They are part of the *Alphaproteobacteria* class, *Rhodospirillales* order, and *Acetobacteraceae* family, with over 100 species across 19 genera now described (1, 2). This group is well-recognized for the fermentation of vinegar [*Acetobacter pasteurianus* (1)], kombucha [*Komagataeibacter* spp. (3–5)], lambic beer [*Acetobacter lambici* (6)], water kefir [*Acetobacter sicerae* (7)], and cocoa [*A. pasteurianus* commercially and *Acetobacter ghanensis/senegalensis* spontaneously (8)], among others. Broadly, AAB acidify lambic beer and vinegar, contribute to flavor, discourage germination in cocoa beans, and produce the cellulose component of a symbiotic culture of bacteria and yeast (i.e. SCOBY) in kombucha (7). AAB also release a variety of metabolic products that have been applied in food, cosmetics, medicine, and other industries (9). These products, including acetic acid (sour flavor and antimicrobial), DHA (dihydroxyacetone; a common sunscreen ingredient), and acetoin (butter-like flavor), result from oxidative fermentation of sugars and alcohols (2, 10). AAB are also found in insect guts, including fruit flies and bees (11), as well as in flowers and fruits (2), all of which are sugar-rich environments (12). Adaptation to these sugar-rich environments may have facilitated success in fermented foods and beverages (7).

Despite their well-recognized importance, there is a limited understanding of the ecology of AAB. One system where AAB are likely important but have largely been overlooked is sourdough starters, which we refer to as sourdough, for brevity. The bulk of sourdough microbiome research has focused on yeast and lactic acid bacteria (LAB). These two functional groups are sufficient to make a sourdough starter (13), although starter microbiomes are often more diverse, ranging from 3 to 10 total species, and include other microbes beyond LAB and yeast (14–17). The “back slopping” method involved in the maturation and maintenance of sourdough starters allows for low-abundance species to become more dominant and for new species to colonize, but factors that allow additional groups to persist are unclear (18). For example, recent work has indicated that AAB are commonly found in sourdough (14, 19–21), but the importance and function of AAB within sourdough remain uncertain as well.

A few studies have begun to investigate the functional roles of AAB in sourdough, but genomic and metabolic characterizations of AAB have been limited to other environments including insect guts (11) and vinegar (22). Common garden experiments with wild sourdough starters suggest that AAB may affect dough rise and aroma (14), but there is limited controlled experimental validation. A strain of *Acetobacter tropicalis* is known to influence the resultant properties of Chinese steamed bread (23, 24). Li et al. (24) found that starters with AAB added had the lowest pH, highest viscosity and elasticity, and had a greater variety of flavor compounds than bread with yeast and LAB or yeast alone. Likewise, the addition of extracted exopolysaccharides (levans and fructans) from AAB to dough resulted in bread that was softer and had more volume, further indicating that the compounds released by AAB can impact emergent traits of sourdough (25). However, insights from the vast majority of ecologically and functionally diverse AAB remain limited.

In addition to their economic and cultural significance, AAB in sourdough also present an opportunity to study the ecological consequences of genomic and strain variation within microbiomes. While it is clear that intraspecies diversity exists across many microbiomes from comparative genomics and metagenomics studies (26–30), there are still surprisingly few studies that have experimentally manipulated strain diversity to understand impacts on microbiome composition and function. Past studies investigating the role of variation at the intraspecies level have found that strain-level differences are associated with microbial community composition and functional traits (31–33), and multiple strains can coexist in an environment due to multiple-niche polymorphism at a small scale (34, 35). As members of microbiomes across a wide range of environments from sourdough to insects, AAB may be generalists, but environmental selection pressures may have resulted in intraspecies diversification. It is also unclear how much strain diversity exists within AAB species in sourdough and other environments and how this diversity contributes to variation in emergent functions of microbiomes. Understanding the functional significance of species and strain diversity within AAB may help reveal novel approaches for managing the functions of fermentations and other AAB-dominated microbiomes.

**Fig 1:**
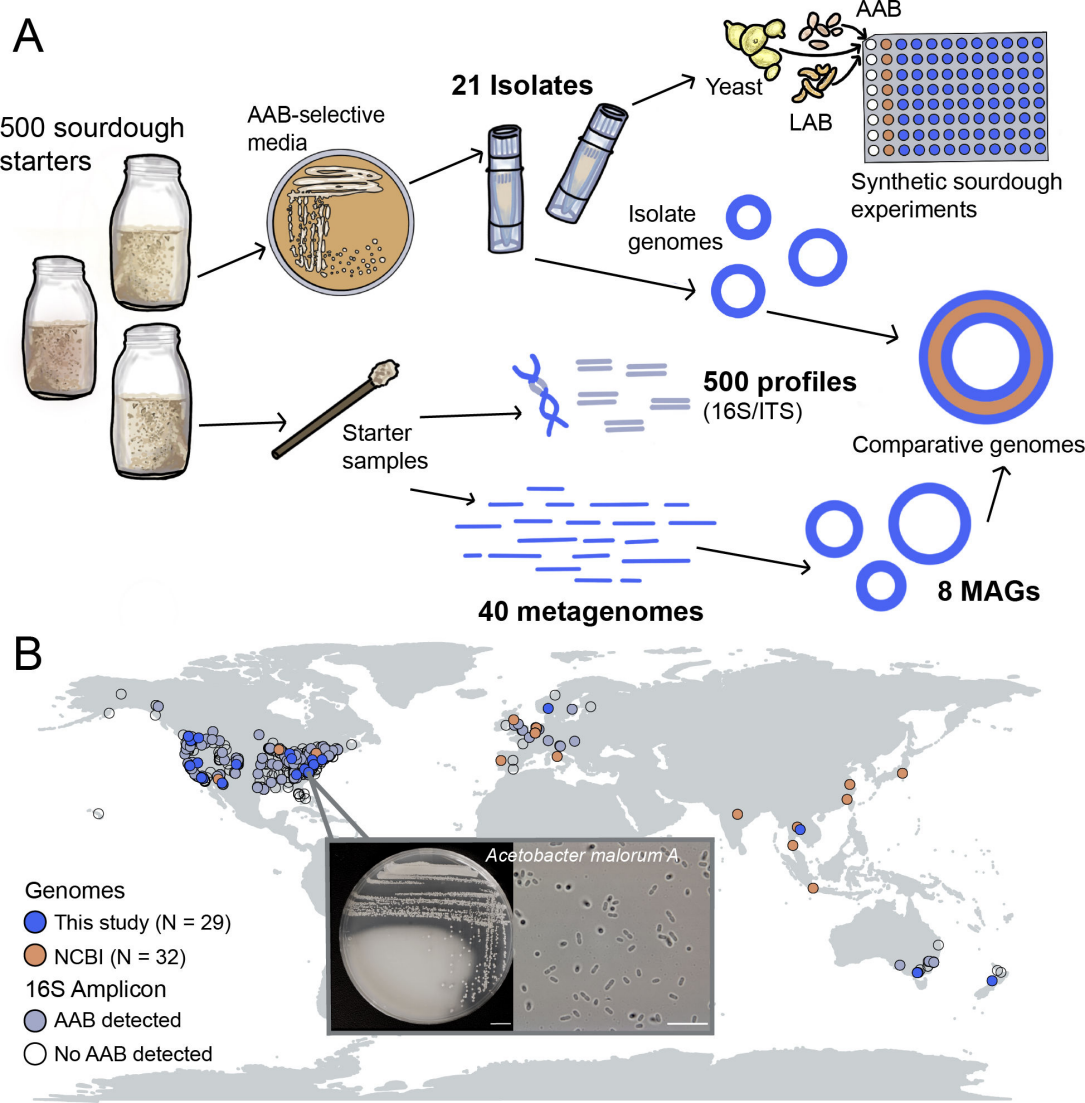
Overview of study design and geographic diversity of AAB. (**A**) Conceptual overview of study data and experiments. Leveraging 16S amplicon data from 500 sourdough starters, we isolated 21 AAB on selective media and obtained corresponding genomes. We obtained MAGs (*N* = 8) from metagenomes (sample *N* = 40) and also included 32 publicly available AAB genomes for a final genome set of 61 for our comparative genomics assessment. We also selected a subset of AAB isolates (*N* = 10) for synthetic sourdough experiments to measure the functional impact of AAB on starter microbiomes. (**B**) AAB were isolated from a set of 500 sourdough samples that were previously described using amplicon sequencing, collected from a global network of community scientists (14). Light blue-gray circles indicate the detection of AAB in 16S rRNA gene amplicon data, and bright blue circles denote the recovery of one or more AAB genomes from the sample. Orange circles denote the set of AAB genomes included from NCBI (National Center for Biotechnology Information), recovered from diverse environmental sources and geographic locations. Popout shows example AAB colonies on plate (s.b. 1 cm) and under the microscope at 100× (s.b. 10 µm).

To shed light on the ecological and functional roles of AAB at multiple levels of genetic similarity in sourdough starter microbiomes, we isolated dominant AAB taxa from a diverse collection of 500 sourdough starters contributed by community scientists from around the world (14). This collection represents 21 strains across 11 species spanning two genera (Fig. 1A). We obtained high-quality draft genomes of all isolates and also obtained eight metagenome-assembled genomes (MAGs) to characterize metabolic pathways and differences in gene content. We assessed if particular functions were enriched in sourdough AAB genomes vs those from other environments broadly across all AAB and within species clusters. We also used our sourdough AAB isolate collection to experimentally determine the function of diverse AAB within the sourdough starter microbiome. Constructing synthetic starter communities with and without various AAB strains, we measured key emergent properties such as acidification and metabolite production. We expect that all AAB will lower the pH of starters due to well-characterized acetic acid production (22) and predict that pH will vary by strain based on corresponding variations in acetic acid production and growth requirements (10, 22). Past research has demonstrated that AAB abundance is strongly correlated with sourdough volatile compound variation (14), but the extent to which particular metabolites differ across AAB strains and species remains unclear. Our work experimentally determines the consequences of an overlooked but functionally important group of microbes in sourdough starters, the acetic acid bacteria, on emergent microbiome function.

## RESULTS AND DISCUSSION

### Acetic acid bacteria are phylogenetically diverse and abundant in sourdough starter microbiomes globally

To determine the ecological distribution and diversity of AAB in sourdough starters, we leveraged a sample collection of 500 sourdough starters collected from a global network of community scientists (14). First, we did a detailed investigation of AAB across all starters (*N* = 500) using a previously sequenced 16S rRNA amplicon data set (14). Then, we obtained AAB genomes from the same starter collection by sequencing isolates and reconstructing microbial genomes from metagenomes. Across the 500 sourdough starters, AAB were present in 29.4% of samples (at ≥1% relative abundance). The mean relative abundance of AAB was 23.3% of the overall bacterial community on average, with up to a maximum relative abundance of 79.5% in a single starter. We detected 26 AAB ASVs (amplicon sequence variants) that span three genera (*Acetobacter*, *Gluconobacter*, and *Komagataeibacter*; Fig. S1; Table S1). In samples where AAB were present, 1.5 AAB ASVs were detected on average.

We next investigated if AAB exhibited consistent patterns of co-occurrence with LAB, yeast, or other AAB, as this might help explain the variable distribution patterns of AAB across starters. We found that LAB including *Schleiferilactobacillus harbinensis*, *Lentilactobacillus kefiri*, and *Furfurilactobacillus rossiae* (Table S2) were enriched (Mann-Whitney, FDR *P* < 0.001 for all) in samples with a high relative abundance of AAB (samples where AAB comprised at least 25% of the overall bacterial community). *S. harbinensis* and *L. kefiri* are common members of water kefir, an acidic beverage that is often populated by AAB, suggesting shared acid tolerance (36, 37). However, the most dominant LAB in sourdough (*Levilactobacillus brevis*, *Lactiplantibacillus plantarum*, *Pediococcus parvulus*, and *Fructilactobacillus sanfranciscensis*) are found similarly with and without AAB. *Pichia mandshurica* was the only yeast significantly associated with AAB-dominant samples. This species is associated with wine spoilage and produces acetic acid as well (38), likely explaining its success in AAB-dominant sourdough samples with higher acidity. *Saccharomyces cerevisiae* made up about 75% of the yeast in both AAB-dominant (76.8%) and non-AAB-dominant samples (75.8%), supporting that AAB can likely survive in most sourdough microbiomes. Within AAB, *Komagataeibacter* and *A. pasteurianus* were frequently detected in the same subset of samples (Spearman’s rho = 0.32, *P* < 0.001), indicating that multiple AAB can co-persist in starters (Table S3).

Leveraging our 16S amplicon data to guide culturing efforts, we isolated 21 AAB strains from 20 sourdough samples. The isolates we cultured span most of the geographic diversity of AAB from our sourdough starter collection (Fig. 1B). AAB were found in starters from 4 continents and 14 countries including the USA, Belgium, China, Thailand, New Zealand, Norway, and India. Reflecting our broader collection, AAB isolates lack representation from South America and Africa and are overrepresented in North America, where we collected the most samples overall. We also added eight MAGs of AAB in sourdough from a set of 40 existing metagenomes (14; Table S4). These MAGs represent more dominant AAB, as the large portion of wheat genome reads impact sensitivity to detect low-abundance AAB. We use both cultured representatives and MAGs for analyses. We did not find any published AAB genomes isolated from sourdough starters based on our search of publicly available databases, so our AAB genomes likely represent a novel collection from sourdough starters. Notably, three of our recovered isolates likely represent putative novel AAB species based on <95% average nucleotide identity (ANI) match to described AAB species, distinct phylogenetic placement, and reported RED (relative evolutionary divergence) values from GTDB-Tk (39, 40; Table S4). We anticipate the number of novel AAB lineages to continue to increase as AAB are further studied in sourdough and other previously overlooked fermentation environments.

**Fig 2:**
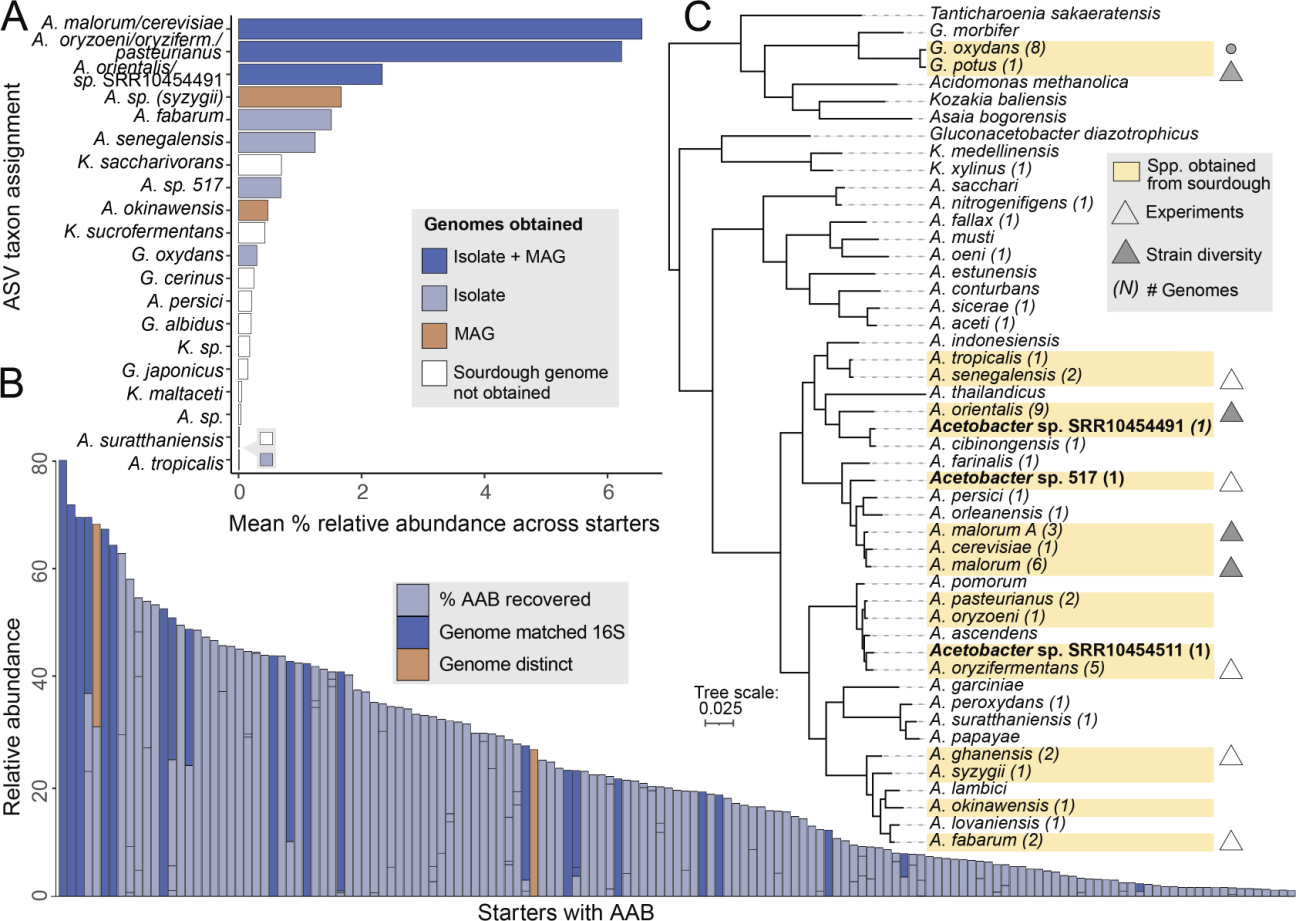
Genomic diversity and abundance of AAB in sourdough starter microbiomes. (**A**) Summary of the mean % relative abundance of AAB by species taxonomic assignments across 500 sourdough starters. Some ASVs could only be assigned to the nearest cluster of species (Fig. S1) due to the low resolution of ASVs. Bar colors indicate the source of genomes obtained (isolate, MAG, both, or no genome obtained from sourdough). (**B**) Summary of the mean % relative abundance of AAB across starter samples. AAB were detected in 29.4% of the 500 starter samples at ≥1%. Breaks in bars represent the relative abundance of each AAB ASV detected in a sample. Dark blue bars denote samples where isolated taxonomy matched the expected identity from 16S amplicon sequencing of the community, and orange denotes samples where a distinct AAB taxon was recovered. (**C**) Representative genome tree of *Acetobacter* species including three putatively novel species (in bold) and other genera including *Gluconobacter* and *Komagataeibacter*, which were also detected in sourdough starter microbiomes. The number of genomes obtained for this study is listed in parentheses (via culturing, metagenome assembly, and download from NCBI). Species with isolates from sourdough or MAGs recovered from sourdough are highlighted in yellow. Triangles represent species that were included in our synthetic sourdough experiments, and filled-in triangles are species that also were included in our assessment of intraspecies strain diversity.

The AAB genomes we obtained represent most of the known phylogenetic diversity of AAB in sourdough starters (Fig. 2). We sought to span the diversity of dominant AAB in sourdough in our targeted culturing efforts. Across the 500 starters, ASVs were assigned to 20 major species clusters as the highest possible resolution. Notably, genomes with as low as 90% ANI were assigned to the same ASV despite representing multiple species, highlighting the limited ability of short-read amplicon sequencing to resolve the genetic diversity of AAB. Through the sequencing and assembly of genomes from both isolates and metagenomes, we obtained representatives from 8 of the 10 most dominant clusters, as well as two representatives from less abundant clusters (Fig. 2A). The most abundant clusters in the 500 sourdough starters were *Acetobacter malorum*/*cerevisiae* and *Acetobacter oryzoeni*/*oryzifermentans*/*pasteurianus* at around 6.5% mean relative abundance. Our isolates reflect the dominant sourdough strains, including *A. malorum*, *A. pasteurianus*, *Acetobacter orientalis*, and *Gluconobacter oxydans,* among others (Fig. 2A; Fig. S2). Although we cultured most of the abundant groups, our efforts did not yield *Komagataeibacter* isolates.

There was high correspondence in the taxonomic identity of isolates obtained from our culturing efforts relative to expected AAB from amplicon sequencing (Fig. 2B), indicating that amplicon-informed targeted culturing is a tractable approach in this system and likely other fermented food systems where most members are readily culturable (41). The sourdough AAB genomes we obtained span much of the broader AAB diversity across environments as well (Fig. 2C), although a couple of clades are notable as they were not detected across our 500 starters. For example, the clade that includes species, such as *Acetobacter aceti*, *Acetobacter nitrogenifigens*, and *Acetobacter sacchari*, does not contain any members detected in ASVs or isolated from sourdough.

### There is extensive variability in gene content and metabolic traits among sourdough AAB

**Fig 3:**
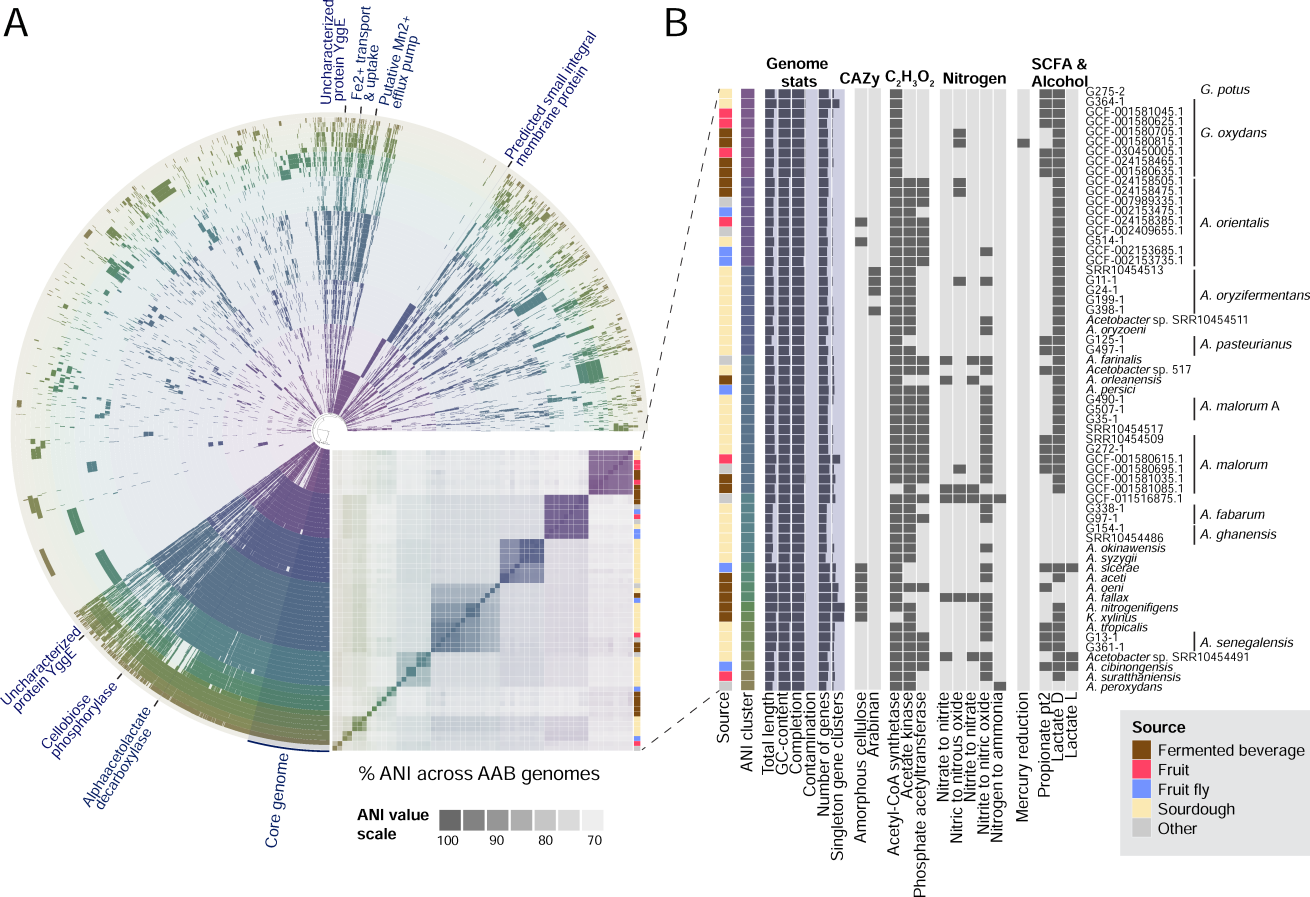
Genomic and metabolic diversity across AAB. (**A**) Pangenome and ANI cluster of 61 AAB genomes. Each ring represents a genome, colored by ANI cluster (Table S5). The core genome across AAB is highlighted, along with a subset of genes functionally enriched in sourdough genomes (for a full set, see Table S8). (**B**) Genome features of the 61 genomes included in the pangenome are reported by isolate source (including fermented beverage, fruit, fruit fly, sourdough, and other), ANI cluster, genome statistics (including % completion and % contamination, Table S4), and metabolic traits that were variable across AAB genomes, including carbohydrate-active enzymes (CAZy) families, acetate pathways, nitrogen metabolism, SCFA i.e. short chain fatty acids, and alcohol production). Genomes are labeled by species. Where multiple genomes per species are included, Refseq ID/original name is given.

To profile variation in gene content and metabolic traits of AAB genomes, we compiled a set of AAB genomes from sourdough and other environments. In addition to our 21 isolate genomes and 8 MAGs, we included 40 high-quality AAB genomes from NCBI isolated from fermented beverages (beer, kombucha, and cider), fruit, fruit flies, and a range of other sources (mud, tree bark, and sewage) resulting in a collection of 61 AAB genomes (Table S4; Fig. S3). The NCBI genomes included three species that we recovered from sourdough (*A. malorum*, *A. orientalis*, and *G. oxydans*) isolated from other sources and other species of AAB (*Acetobacter fallax*, *Acetobacter lovaniensis*, *A. aceti*, and *Komagataeibacter xylinus*). Across AAB, differences in ANI ranged from 70% (across genera), a noted ANI gap of 90%–95% observed in closely related species clusters, and 96%–98% within species (Table S5). Across all genomes (*N* = 61), we detected 11,596 unique gene clusters. Of these, 909 represented core gene clusters that were present in >95% of genomes (7.8%). The other 10,687 gene clusters were only detected in a subset of genomes and represent both species-specific clusters and other accessory genes that were variably detected (Fig. 3A; Table S6).

We detected notable variation in AAB metabolism within and between species (Fig. 3B; Table S7) and source environment (Table S8). For example, genes for cellulose production were detected in several fermented beverage genomes within *A. aceti* and *A. nitrogenifigens*. In contrast, cellulose production was detected in only one sourdough genome, *A. orientalis* (Fig. 3B; Table S7). In kombucha, cellulose production is a favored trait of *A. nitrogenifigens* to form the pellicle used to inoculate future batches (42, 43). In contrast, cellulose production may be a disfavored trait in sourdough due to interference with structural properties. Arabinan utilization genes were detected in four out of five *A. oryzifermentans* isolated from sourdough. Arabinan is a plant polysaccharide, and the ability to break it down likely has functional consequences within the sourdough starter microbiome, which is fed by wheat flour. For all gene and pathway data presented in Fig. 3B, including acetate, nitrogen, short-chain fatty acid, and alcohol metabolism, see Table S7.

### Intraspecies variation may favor the persistence of AAB with key traits related to resource utilization in the sourdough environment

**Fig 4:**
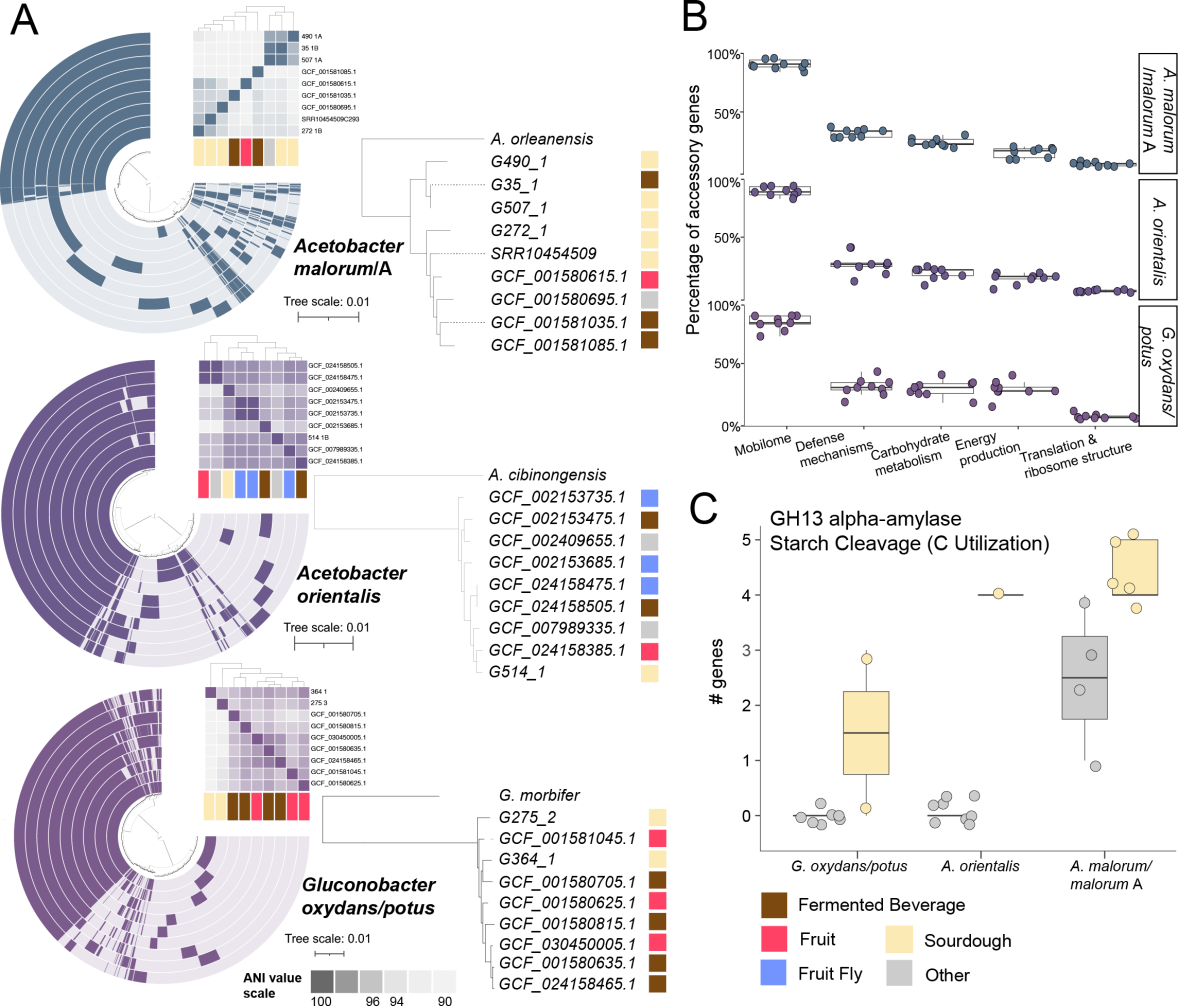
Diversity within AAB species. (**A**) Strain pangenomes with ANI heat map (Table S5) and corresponding genome trees (SpeciesTree) of nine strains each of *A. malorum/*A, *A. orientalis*, and *G. oxydans/potus*. Pangenomes and trees are annotated by the isolation source. (**B**) Percentage of genes in the accessory genome within a subset of functional categories (mobilome, defense mechanisms, carbohydrate metabolism, energy metabolism, and translation) across the three species highlighting strain diversity. (**C**) Boxplot highlights GH13 alpha-amylase (starch cleavage) which was found to be enriched in sourdough starter vs other environments in multiple strains of the same species.

To determine if any genes were enriched in sourdough relative to other environments at the strain and species cluster levels (where % ANI amongst members ranged from 90% to 99%), we next focused our analyses on a subset of three species for which we had both sourdough and non-sourdough genome representatives: *A. malorum*/*malorum* A, *A. orientalis*, and *G. oxydans/potus*. We note that both *A. malorum* A and *G. potus* were recently reclassified as distinct species from *A. malorum* and *G. oxydans*, respectively. We first performed pangenomic analyses on nine genomes within each of the three species clusters. The total gene clusters ranged from 3,998 (*A. orientalis*) to 6,207 (*A. malorum/malorum* A; Fig. 4A; Table S6; Fig. S4). On average, species core genomes comprised ~40% of gene clusters, whereas the other 60% of gene clusters were accessory.

There was no clustering of overall genome content by either isolation source or geographic location within these species (Fig. 3A). This could be because many of the AAB in sourdough starter microbiomes are successful generalists adapted to a wide range of environments rather than one specific niche. They may have a nomadic lifestyle similar to some species of LAB such as *L. plantarum* (44, 45) and the AAB species *A. senegalensis* (46). Alternatively, the inclusion of more AAB genomes from sourdough and other environments may reveal signatures of adaptation that are environment specific. Despite an overall lack of clustering by geography or isolation source, we found potential gene functions that were enriched in sourdough across multiple strains. GH13 alpha-amylase, involved in starch cleavage, was enriched in sourdough with the presence of 45% of strains relative to 28% of strains not isolated from sourdough (Kruskal-Wallis FDR-corrected *P* < 0.01; Fig. 4C; Table S9). Starch is a major component of wheat, so this could confer a competitive advantage to AAB in sourdough vs other environments (47, 48). Although limited by the availability of high-quality AAB genomes from other environments, our analyses start to shed light on the metabolic traits that may favor the persistence of AAB in the sourdough environment.

While gene clusters of unknown function were much more frequent in accessory genomes, functionally annotated accessory genes largely fell into categories that may have relevance to success in sourdough. On average, 61.0% of genes were annotated with a known function (COG database) in accessory vs 87.3% in core (Table S6). Although expected, this underscores the difficulty in determining the functional or ecological significance of gene content variation. Mobilome genes, related to transposons and prophages, made up the largest percentage of accessory genes (Fig. 4B; Fig. S5; Table S10). *A. pasteurianus* has been known to have a high level of genetic variability particularly linked to the mobilome, with 9% of genes encoding transposases (49). Twenty-two carbohydrate-active enzymes (CAZy) that may be relevant to success in sourdough were found in plasmids and carried by prophages in the genomes we recovered from sourdough, including the GH13 alpha-amylase significantly associated with sourdough genomes (Fig. S6). Notably, an average of 24% of genes related to carbohydrate utilization were detected in accessory genomes across the three species clusters, underscoring the variability in microbial traits related to nutrient acquisition. Accessory carbohydrate utilization genes included 5-carboxyvanillate decarboxylase LigW, involved in lignin degradation, and alpha-galactosidase. The high intraspecies variation in resource utilization genes may impact the establishment success and persistence of the AAB strain within the sourdough microbiome.

### Synthetic common garden experiments reveal distinct species and strain level effects of acetic acid bacteria on overall microbiome function

**Fig 5:**
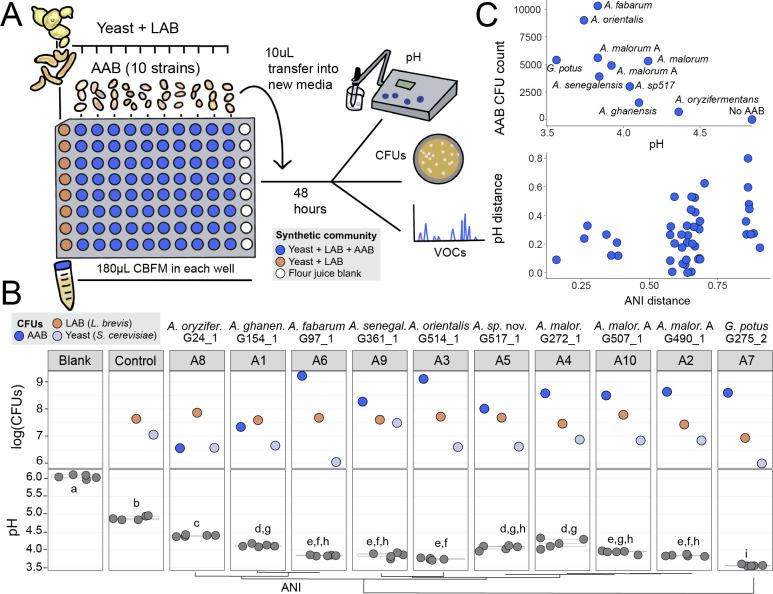
Sourdough starter acidification is determined by differences in genus, species, and strain-level variation in AAB. (**A**) Experimental design of synthetic communities. Ten isolates of AAB were selected as treatment groups and added to a background starter community of yeast (*S. cerevisiae*) and LAB (*L. brevis*). We also included a yeast + LAB only control and cereal-based fermentation medium (CBFM) blank where no microbes were added. All isolates were added in 5 µL at a total density of 20,000 colony-forming units (CFUs). (**B**) The total abundance of each member of SynCom129 measured via CFUs and plotted with log10 (top); acidification measured via pH from the liquid CBFM and reported at the end of 4 days of incubation and transfers (*N* = 5 replicate synthetic communities; dots represent individual observations and box plots summarize the distribution). (**C**) Plots of the relationships between AAB CFU count and emergent microbiome pH (top) and ANI vs pH distance (bottom) from SynCom129.

To understand the consequences of acetic acid bacteria on the assembly and emergent function of sourdough starter microbiomes, we constructed replicate synthetic sourdough starter communities, varying only the AAB strain across treatments (Fig. 5A). We proxy microbiome function by pH and volatile organic compounds (VOCs) and also measure the abundance of community members. We selected 10 AAB strains that represented the breadth of phylogenetic diversity observed and included intraspecies diversity within the *A. malorum* cluster. We qualitatively observed morphological differences in size and color across the 10 strains (Fig. S7). For example, *Acetobacter sp. nov*. 517 and *G. potus* colonies were smaller, whereas *A. malorum* and *A. malorum* A were the largest. This could be due to differences in starting concentrations, nutritional requirements, or growth rates. Most colonies were white to pink in color, but *A. fabarum* had yellow colonies (Fig. S7). *A. orientalis* was observed to produce a biofilm, likely bacterial cellulose, or another exopolysaccharide. Cellulose is produced by many AAB including those isolated from kombucha (50, 51), but amorphous colonies due to cellulose production were not commonly observed in the AAB strains isolated from sourdough.

For our synthetic experiments, we selected a LAB (*L. brevis*) and yeast (*S. cerevisiae*) as the base community, isolated from wild starter S_129 (resulting in “SynCom129”) varying only the AAB strain added. We also included a media-only blank {all synthetic communities were grown in cereal-based fermentation medium [CBFM; (14)], a liquid-based cereal fermentation media} and a YL-only control with only yeast and LAB present. This design resulted in 12 distinct treatments. For the number of replicates run for each assay (i.e., pH, VOC production), see Table S11. After incubating for 48 hours, transfer to fresh media, and incubation for another 48 hours (4 days total), we measured the resultant community structure and emergent functions across treatments. We assessed the persistence of all starting members and emergent functions of the resultant starter including acidification (pH) and VOC profiles. We selected a second background community of *L. brevis* and *S. cerevisiae* (SynCom361) isolated from a second wild sourdough (S_361) to confirm the trends we observed with pH and persistence analyses.

In both synthetic communities, all 10 AAB strains persisted across replicates. SynCom129 was isolated from a starter without AAB, suggesting that AAB are likely able to establish and persist in a range of starters. While yeast and LAB colony-forming units (CFUs) counts stayed fairly consistent, AAB CFU counts varied greatly by strain, indicating the potential differential success of strains in the synthetic communities. Higher AAB CFU counts were significantly correlated with lower pH (Spearman’s rho = −0.83, *P* = 0.002), while LAB and yeast CFU counts were not correlated (Fig. 5C; Table S14). The pH was also correlated with AAB ANI (*P* = 0.055; Fig. 5C; Table S14). These results suggest that AAB density and differences in genome content may play a role in modulating starter acidification. The yeast and LAB persisted in every condition in SynCom129, but in SynCom361, the yeast sometimes failed to persist. This could be due to a mismatch of nutritional requirements but also may reflect inhibition by the AAB. We observed yeast dropout in one condition where yeast appeared glued in EPS (exopolysaccharides) from *A. orientalis* (Fig. S8).

All AAB treatments acidified the starter environment, and the extent was strain-specific (Fig. S9). In SynCom129, in comparison to the YL-only control, AAB overall decreased the pH of the sourdough microbiome by 18.5% in a 4-day period. The starting pH on day 0 of CBFM was around 6.26. By day 2, communities had begun to acidify the starter environment. The average pH of communities with AAB was 4.20 (*N* = 30, SD = 0.30), and the average pH for YL-only was 5.64 (*N* = 3, SD = 0.03). On day 4, all communities with AAB had significantly lower pH from YL-only and CBFM blank (*P* < 0.0001; Fig. 5B; Table S14). The average pH of communities with AAB on day 4 was 3.94 (*N* = 50, SD = 0.23), while the average for YL-only was 4.84 (*N* = 5, SD = 0.05). Significant acidification was also observed in SynCom361 on day 4 between AAB and non-AAB communities (*P* < 0.0001) despite yeast dropout in some replicates (Fig. S9). This is consistent with studies from other fermented foods that have shown that AAB lower the pH of their environment (6, 7). The release of acetic acid and a corresponding decrease in pH may inhibit the growth of other species and aid in AAB persistence (52). While pH was lowered in all AAB treatments, there were significant differences between species, including between strains of closely related species. *A. oryzifermentans* (average 4.36, SD = 0.02) and *G. potus* (average 3.56, SD = 0.02) were significantly different from all other AAB strains. The two *A. malorum* A strains did not significantly differ from each other, while the strain of *A. malorum* differed from only *A. malorum* A 490. This finding underscores the importance of species and strain-level diversification on emergent function.

**Fig 6:**
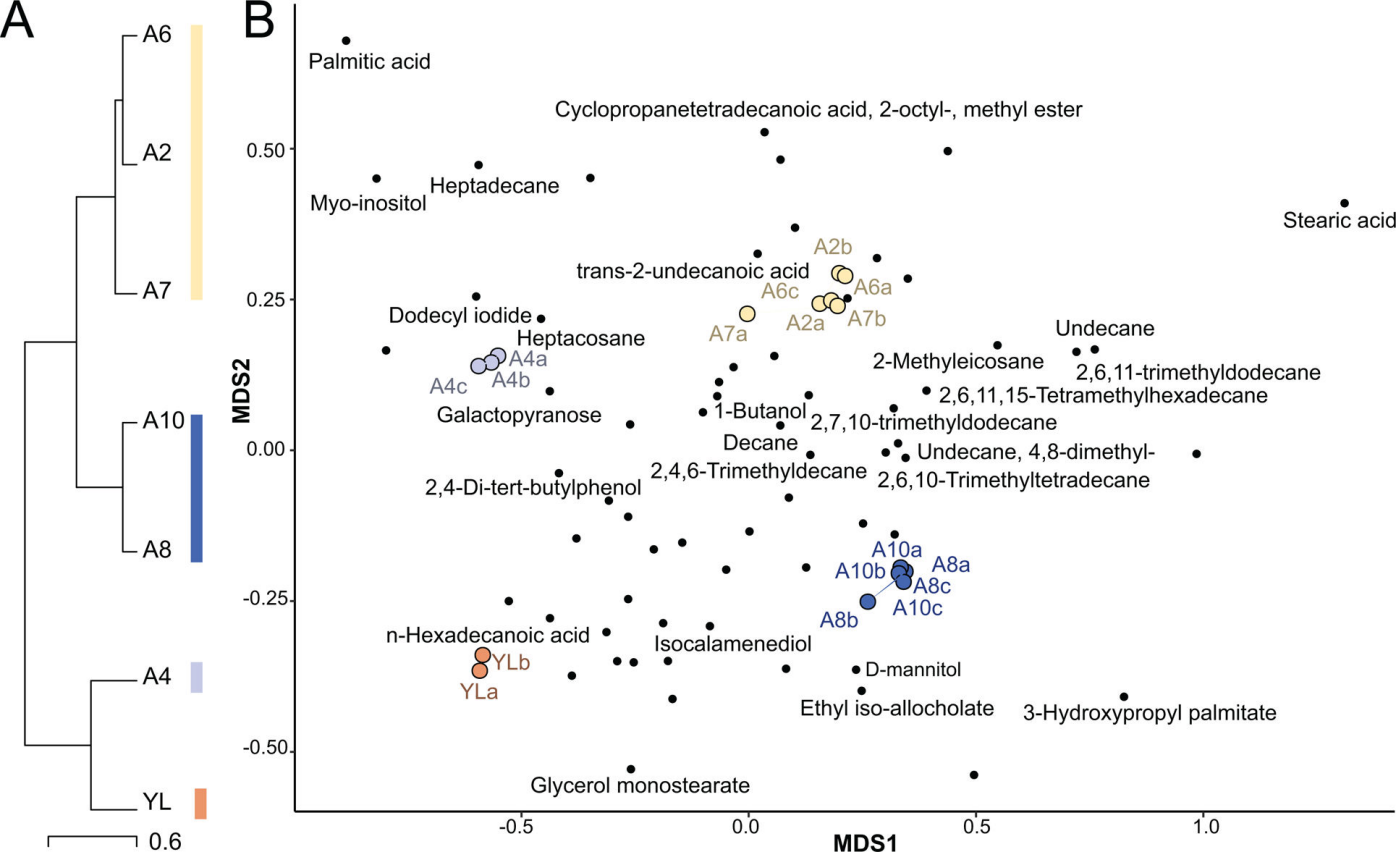
Sourdough VOCs are associated with microbial community type. (**A**) Dendrogram of VOC hierarchical clustering resulting in four sample clusters. (**B**) Superimposed NMDS plots of liquid sourdough communities and VOCs by community. The four main clusters have been colored. VOCs have been labeled when significantly different between clusters.

Sourdough VOCs significantly differed by starter community members. We analyzed the VOCs from seven different communities frozen on day 4, including six AAB treatments and a yeast-LAB-only control (Table S12). VOCs in the YL community significantly differed from all AAB communities (*P* = 0.008; Table S14), and the AAB communities formed three clusters (A4 alone, A2/A6/A7, and A8/A10; Fig. 6A) with distinct VOC profiles (*P* = 0.01; Fig. 6B; Table S14). Clusters were not associated with phylogeny. Notably, despite 97.2% similarity in ANI, *A. malorum* A 507 and *A. malorum* A 490 were in different clusters. A variety of compounds differed between the clusters (Table S13; Fig. 6B). Stearic acid (*P* = 0.02; AAB-associated clusters) and palmitic acid (*P* = 0.02; higher in the no-AAB controls and cluster A4) have both been previously detected in ferments including Tarhana [a Turkish wheat and yogurt-based fermented food (53)]. We also detected compounds previously linked to flavor formation in sourdough including decanal, which is associated with vinegar and a sweet, citrusy flavor (54), and L-arabitol, which is associated with sweet flavor (55). Both were more abundant in samples with AAB, although not significantly (Table S13). Our results suggest that strain-level variation in AAB members can impact the sensory properties of the resulting community.

### Conclusions

Our study represents a genomic and ecological characterization of AAB isolated from sourdough starters and demonstrates the utility of synthetic consortia to determine the functional consequences of specific microbiome members. Our genomic analyses begin to uncover the diversity in AAB genome content and suggest that sourdough AAB may be enriched in specific gene functions that facilitate success in the sourdough environment. These analyses also suggest that community-wide emergent functions can be affected by differences in gene content resulting from intraspecies diversification. Our comparative genomic analyses were limited, however, by the number of high-quality and publicly available AAB genomes from other environments. Increasing genomic representation from this group of economically and culturally significant bacteria will continue to yield new insights on their metabolic diversity and the ways in which they may be adapted to diverse sugar-rich environments. Likewise, obtaining closed genomes using approaches such as long-read and hybrid sequencing will facilitate a more resolved understanding of the location of key regions and genes of interest and the opportunity to study population-scale dynamics.

Our synthetic experiments demonstrate that phylogenetically and metabolically diverse AAB can persist in common sourdough microbiomes. We also determine the impact of AAB species and strain-level diversity on emergent sourdough functions including acidification and volatile profiles. Notably, we found that intraspecies variation in *A. malorum* resulted in significantly different community VOC profiles of synthetic starters. These findings have direct relevance to industrial and home-bakers considering flavor profiles and other features of sourdough bread. A clear next step is to extend these results and assess how AAB may impact functional consequences in baked sourdough bread. Similarly, our synthetic experiments were performed using an *S. cerevisae* and *L. brevis* background community. Although this pairing represents one of the most dominant sourdough LAB and yeast combinations, it will be important to determine if AAB have similar functional consequences in other common sourdough types such as *F. sanfranciscensis* and *Kazachstania humilis*. An exciting avenue for future research is using tools such as interaction screens, RNA-seq, and metabolomics to shed light on what molecular-level mechanisms underlie the observed community-wide functional shifts.

## MATERIALS AND METHODS

### 16S rRNA amplicon analysis of AAB in sourdough

For our amplicon-based analyses of the distribution of AAB across 500 starters and their associations with LAB and yeasts, we used previously sequenced 16S and ITS rRNA gene sequence data from reference (14). In brief, DNA was extracted from 2 mL starter subsamples with the Qiagen PowerSoil DNA extraction kit and sequenced with Illumina MiSeq at the University of Colorado Next-Generation Sequencing Facility. Sequences were processed with the DADA2 pipeline, resulting in taxonomic assignments and ASV tables of relative abundances. We calculated the mean relative abundance of AAB in starter samples and also assessed co-occurrence of AAB with other starter members. First, we used Spearman’s correlations to test if any AAB ASVs significantly co-occurred with any LAB, yeast, or other AAB ASVs. We only included ASVs that had at least a mean relative abundance of 0.1% (Table S3). We also tested whether particular LAB or yeast tended to be enriched in AAB dominant samples using Mann-Whitney tests (Table S2) with FDR-adjusted *P*-values. We defined AAB dominant as samples where the AAB comprised at least 25% of the overall bacterial community.

### Isolation and sequencing of acetic acid bacteria strains

We leveraged the previously sequenced 16S rRNA gene amplicon data of 500 sourdough starters described above (14) to selectively target starter samples that had a high relative abundance of AAB and to capture a broad range of phylogenetically diverse AAB in our culturing efforts. We obtained isolates of 21 acetic acid bacteria, most of which were abundant in samples based on the amplicon data and were isolated from frozen (−80°C) sourdough starter samples. Subsamples were plated onto selective GYCA medium (per liter, 30 g glucose, 5 g yeast extract, 3 g peptone, and 15 g agar) with 10 g CaCO_3_ supplemented with 25 mL natamycin (21.6 mg/L), and subsamples that had amplicon hits to *Gluconobacter* were plated onto Carr medium supplemented with natamycin (21.6 mg/L) (56). To construct synthetic starter communities, we also obtained isolates of *L. brevis* (LAB) which was plated onto Lactobacilli MRS agar (Criterion) with natamycin (21.6 mg/L) and of *S. cerevisiae* which was plated on yeast potato dextrose medium with chloramphenicol (50 mg/L). Distinct morphologies were cultured from each sample, and we used Sanger sequencing to determine general taxonomic identity with the 16S rRNA primer sets 27F/1492R for bacteria and ITS1F/ITS4R for yeast. Isolate cultures were stored in 15% glycerol at −80°C.

To sequence isolate genomes, DNA was extracted from grown colonies using ZymoBIOMICS DNA extraction kits, with bead bashing for cell lysis (Seqcenter, PA). Following extraction, sample libraries were prepped with the Illumina DNA prep kit with 10 bp dual indices (IDT) and sequenced on an Illumina NovaSeq 6,000 resulting in 151 bp, paired-end reads. We targeted 200 Mbp per sample (e.g., isolate). After sequencing reads were demultiplexed and adapters were trimmed using bcl-convert v4.1.5, we obtained an average of 5,676,503.62 paired-end reads per sample (ranging from 3,703,604 to 10,182,868) and an average % bp >Q30 of 91.096%.

To assemble high-quality draft genomes from raw reads for each isolate in KBase (57), reads were trimmed to remove low-quality bases at both ends with BBTools v38.22 (58), and low-complexity reads were filtered out with PRINSEQ (59). Read quality was assessed with FastQC v0.11.0 prior to assembly with SPAdes v3.15.3. For a few genomes (*N* = 6), the metaSPAdes assembly algorithm (60) resulted in a higher-quality assembly. All genomes obtained were high quality, with ≥93.53% complete and <3.23% contaminated, and the median number of contigs was 29. After assessing the genome quality of isolates with CheckM v1.0.18 (61), genomes were taxonomically classified with GTDB-Tk v1.7.0 (39, 40). We also used GTDB-Tk to assess the taxonomic novelty of isolates based on reported ANI and RED values (39, 40). Genomes that did not fall within 95% ANI of existing reference genomes are considered putatively novel species. We also placed all genomes phylogenetically using KBase SpeciesTree v2.2.0 (57), which constructs species trees from a set of 49 universal core genes defined by COG gene families to determine relatedness. Assemblies were annotated with Prokka v1.14.5 (62) and re-annotated in anvi’o [(63); see comparative genomics section below] with anvi-run-hmms and anvi-run-ncbi-cogs (64).

### Inclusion of additional genomes from sourdough metagenomes and NCBI

To complement the AAB genomes that we obtained by culturing from sourdough starter samples, we also assembled genomes from 40 shotgun metagenomic samples, a subset of the 500-starter collection (14), deposited in NCBI under Bioproject PRJNA589612. After quality filtering with BBDuk (65) and filtering reads that aligned to the bread wheat genome (IWGSC CS RefSeq v2.1) using bowtie2 (66), we used metaSPAdes v. 3.15.5 (60) to assemble contigs. Next, contigs were placed into genome bins with both VAMB (67) and Metabat v. 2.15 (68), and then DasTool v. 1.1.5 (69) was used to select the best bins for each sample. CheckM v. 1.2.2 (61) was used to score the bins. Next, bins from all samples were collected together, and dRep v. 3.4 (70) was used to make a dereplicated set with the highest-quality representatives at 95% ANI cutoff to represent species-level genomes (parameters that deviated from defaults included: completeness, contamination, and coverage, which were respectively set to 50.0, 10.0, and 0.3). GTDB-tk v. 2.1.1 (39) was used to taxonomically classify the full set of dereplicated bins. We then filtered the bins based on taxonomy to only include AAB bins (*N* = 10 out of 30). We also built a genome tree of isolates and MAGs to identify any redundancy between genomes and MAGs. Where overlap was detected, the isolate was selected for downstream analysis, and the MAG was excluded. This resulted in a final set of eight MAGs.

To contextualize our AAB sourdough genomes within the broader context of AAB genomic diversity and to assess if any gene content was enriched in sourdough environments, we also obtained publicly available AAB genomes from NCBI to include in our analyses. We found no publicly available genomes from *Acetobacter*, *Gluconobacter*, or *Komagataeibacter* isolated from sourdough on the JGI GOLD genomes database. For an analysis of strain diversity, we added genomes from NCBI to a set of genomes we obtained from sourdough from *A. malorum* (recently split with *A. malorum* A, ~90% ANI)*, A. orientalis*, and *G. oxydans* (recently split with *G. potus*, ~94% ANI) to compile a set of nine genomes within each species (19 genomes total from NCBI with at least 89% completion and less than 6.88% contamination). These three species were chosen based on the availability of multiple genomes from a variety of sources, as most AAB species have only one to a few genomes sequenced, and *A. malorum* genomes were targeted as part of the most common cluster of AAB genomes from sourdough. We were limited to nine genomes per species cluster by the poor quality of publicly available genomes as well, even after setting a reasonable cutoff for percent completion and contamination. We sought to include a representative set of genomes from across the tree of *Acetobacter*. We constructed a genome tree of all species of *Acetobacter* listed on NCBI Taxonomy using SpeciesTree - v2.2.0 with a reference genome from each species (Fig. 2C) and downloaded 12 additional *Acetobacter* genomes from across the tree that we did not recover from sourdough, along with one species from an additional genus, *K. xylinus*, as an outgroup. We targeted genomes that were at least 90% complete with <5% contamination and only included genomes that had available source and location information. When source data did not include latitude and longitude, the center point of the country or city was chosen using latlong.net.

To assess how well the genomes we recovered in this study represent the known diversity of AAB in sourdough, we leveraged our 16S rRNA gene sequencing of 500 sourdough starters. First, we assigned ASV taxonomy using a phylogeny-guided approach. We extracted full or partial 16S rRNA gene sequences from isolate, MAG, and NCBI genomes using ContEst16S (71) and aligned the sequences along with our partial 16S ASV amplicon sequences (resulting in a total of 197 AAB sequences included in the phylogeny) using the SINA aligner (72). After gap trimming (gap threshold = 0.2), we constructed a phylogenetic tree using RAxML-HPC2 on XSEDE (Fig. S1). ASV taxonomic assignments were then made to the nearest genome representative. When an ASV was close to genomes from multiple species due to limited phylogenetic resolution, we assigned a cluster identity (such as the *A. malorum—cerevisiae* cluster). We note that this phylogeny is based on short-read data and is a useful way to improve taxonomic assignments but is not intended to accurately represent phylogenetic relationships.

### Comparative genomic analyses

In total, we included 61 AAB genomes in our cross-environment comparative analysis (21 isolates, 8 MAGs, and 32 from NCBI). All 61 genomes were annotated with DRAM (73) to additionally profile a suite of microbial traits related to categories including short-chain fatty acid and alcohol conversions, nitrogen metabolism, and CAZy. We display a subset of these functions that are differential across AAB that were annotated and distilled using DRAM (73; Fig. 3B; Table S7). Detailed annotation files from DRAM are available on Figshare DOI 10.6084 under 10.6084 /m9.figshare.25403599. We also constructed a pangenome of all 61 genomes in anvi’o (minbit 0.5, mcl-inflation 6, and min-occurrence 2) (74, 75) and computed ANI using pyANI (76). We annotated genomes by source (sourdough or other) and tested for functional enrichment by source and species using the command “anvi-compute-functional-enrichment-in-pan” (77), a tool that finds functions that are enriched in the specified group and relative to genomes outside the group. We used the same approach to build the three species-level pangenomes for *A. malorum/malorum* A, *A. orientalis*, and *G. oxydans/potus* with corresponding ANI and functional enrichment analyses, using mcl-inflation 10 and no min occurrence. We also identified viruses and plasmids within the genomes recovered from sourdough using geNomad and ran DRAMv on viral regions and DRAM on plasmids to annotate genes in those regions (Fig. S6; Table S10).

### Construction of synthetic starter communities

For synthetic sourdough starter experiments, we used a CBFM that approximates the dough environment, as described in reference (14). Briefly, to make the media, whole wheat and all-purpose flour (Bob’s Red Mill) were mixed with water in a 1:1:9 ratio, centrifuged (3,000 rpm) to pellet flour particles, and then filtered through a 0.20 µm filter to remove microbial cells from the filtrate. To measure the effects of AAB on starter microbiome composition and function, we constructed two LAB and yeast communities: SynCom129, LAB *L. brevis* strain 129 plus yeast *S. cerevisiae* strain 129, and Syncom361, *L. brevis* strain 361 1A plus *S. cerevisiae* strain 361. These two species are dominant in sourdough starters and are frequently found together in the same starter (14). Additionally, this LAB/yeast pairing is commonly found in starters with AAB (the case for strains 361) and also in starters without AAB (the case for strains 129). We then selected a subset of 10 AAB isolates corresponding to 10 treatment groups that spanned the breadth of phylogenetic diversity we obtained: two genera (*Acetobacter* and *Gluconobacter*), eight species (*G. potus*, *A. malorum*, *A. sp*. 517, *A. orientalis*, *A. senegalensis*, *A fabarum*, *A. ghanensis*, and *A. oryzifermentans*), and three strains within *A. malorum/malorum* A. We also included a “no AAB” treatment control group (only LAB/yeast) and a blank with just CBFM.

In 96-well plates, approximately 5 µL of 4,000 CFUs/μL were inoculated into 180 µL of CBFM and 5 µL PBS resulting in a total volume of 200 µL (10 µL PBS was added for no AAB control, and 20 µL PBS was added for blank). For each condition (10 AAB strains + 1 no AAB + 1 CBFM blank), we constructed eight replicate communities, resulting in 96 synthetic communities in total for each SynCom. Communities were incubated aerobically at room temperature on the bench, and 10 µL of the resultant culture was transferred 48 h after initial inoculation into 190 µL of fresh CBFM. At this point, wells were tested for presence/absence, and pH was collected. After the cultures grew for another 48 h (4 days in total), we harvested the experiment: three wells from each condition of the SynCom129 plate (180 µL/ well) were immediately frozen at −80°C for metabolite analyses, and 100 µL from one well of each condition was subsampled to determine total abundance of each member (AAB, LAB, and yeast) by serial dilution and plating on selective media at 10^−4^ and 10^−5^ to count CFUs. Additionally, pH was assessed *in situ* from the remaining unfrozen communities in SynCom129 and the corresponding communities in SynCom361, and one well of each condition was streaked for presence/absence (see below).

### Assessment of metabolites and acidification

All statistical tests were executed in the R environment (version 2023.06.0 + 421, R Core Team). To measure the overall acidification of sourdough starter communities, the pH of each synthetic sourdough community (*N* = 216, excluding those that were sampled for metabolomics to avoid contamination) was taken with a pHenomenal pH meter (VWR) with an MI-410 Combination microprobe (Microelectrodes, Inc.) at the conclusion of the experiment. For our AAB synthetic community experiments, we used analyses of variance to determine the effects of AAB on community pH, with AAB strain identity as the independent variable. We included a no AAB control (i.e., *L. brevis* and *S. cerevisiae* only) that we refer to as the “background community” and also a CBFM-only blank. We then used Tukey’s post hoc significance testing to assess the significance of pairwise differences (Fig. 5B). To assess if pH was correlated with the density of AAB, LAB, or yeast, we used Spearman’s correlations (Fig. 5C). To determine if either the observed pH values or CFU counts were correlated with AAB phylogenetic distance, we used mantel tests (method = spearman) to correlate the pairwise distances (pH distance metric used was euclidean), with 9,999 permutations (Fig. 5C).

To prepare samples for GC/MS analysis, 180 µL aliquots were harvested from the final communities excluding the control and immediately frozen at −80°C. Samples were shipped to Creative Proteomics (Shirley, NY), and upon arrival were thawed on ice, and 0.1 mL of each of the 21 samples was transferred to a tube with 0.3 mL 80% methanol solution and 5 µL ribitol (5 mg/mL). The tubes were vortexed for 60 seconds and ground for 180 seconds at 60 Hz, sonicated for 30 minutes at 4°C, and centrifuged at 12,000 rpm and 4°C for 10 minutes (Eppendorf). Two hundred microliter of supernatant was transferred into a new tube, dried under nitrogen, and then added to 35 ul O-Methylhydroxylame solution (Merck), vortexed for 30 seconds, and incubated at 37°C for 90 minutes. The extractives were derived with 35 µL BSTFA, reacted at 70°C for 60 minutes, and kept at room temperature for 30 minutes until analysis. Samples were analyzed on a DB-5 column (60 m × 0.25 mm × 0.25 µm, Agilent). The detector and injector temperatures were 280°C. The oven temperature was held at 70°C for 5 minutes then raised to 200°C at a rate of 10°C per minute, then raised to 280°C at 5°C per minute and held for 10 minutes. Helium was used for column carrier gas at a constant flow rate of 1 mL/min, and split (20:1) injection mode was used. The temperature of the ion source was 230°C, the electron impact mass spectra were recorded at 70 eV ionization energy, and the GC-MS (gas chromatography–mass spectrometry) analysis was carried out in 30–550 mass range scanning mode (Thermo Scientific ISQ 7000).

To analyze the variation of VOCs across the samples sent for GC/MS analysis, any duplicated compound reads were removed, only keeping the highest similarity matches of each compound. Compounds related to plastic components/degradation, human metabolites, or low-similarity compounds were also removed as background or contamination. Then, distance matrices were calculated and ordinated of the VOCs and samples (Fig. 6B). Four outlier samples were removed prior to downstream analyses (YL_C, A2_C, A6_B, and A7_C). To assess the impact of AAB treatment on overall community VOC profiles, we ran PERMANOVA models with treatment (AAB strain) and AAB presence (vs yeast-LAB only) as predictors. We identified four clusters of samples using hierarchical clustering (method Ward D2) and plotted the resulting dendrogram (Fig. 6A). We used Kruskal-Wallis tests (with FDR corrections for multiple comparisons) to assess if any VOCs were significantly enriched by AAB presence, treatment, or cluster.

## Data Availability

Raw reads and assemblies for all genomes generated in this study have been deposited in the NCBI Sequence Read Archive in BioProject PRJNA1095457. Annotation, pangenome files, and DRAM output have been deposited to Figshare DOI 10.6084 under (10.6084/m9.figshare.25403626, 10.6084/m9.figshare.25403620, and 10.6084/m9.figshare.25403599, respectively). Code is available on GitHub at https://github.com/hbrappaport/aab_manuscript_code.git. .

## SUPPLEMENTAL MATERIAL

Supplemental figures msystems.00537-24-s0001.docx Fig. S1 to S9.

Captions msystems.00537-24-s0002.docx Captions for Tables S1 to S14.

Supplemental tables msystems.00537-24-s0003.xlsx Tables S1 to S14.

ASM does not own the copyrights to Supplemental Material that may be linked to, or accessed through, an article. The authors have granted ASM a non-exclusive, world-wide license to publish the Supplemental Material files. Please contact the corresponding author directly for reuse.
