# Supplementary material for: Genomics and synthetic community experiments uncover the key metabolic roles of acetic acid bacteria in sourdough starter microbiomes
Source: mSystems. 2024 Sep 17;9(10):e00537-24. doi: 10.1128/msystems.00537-24 (PMC11498085; doi:10.1128/msystems.00537-24)
Supplement: Supplemental figures — Fig. S1 to S9. [file msystems.00537-24-s0001.docx]

**­Supplemental Figures, *Genomics and synthetic community experiments uncover the key metabolic roles of acetic acid bacteria in sourdough starter microbiomes*:**

**­­
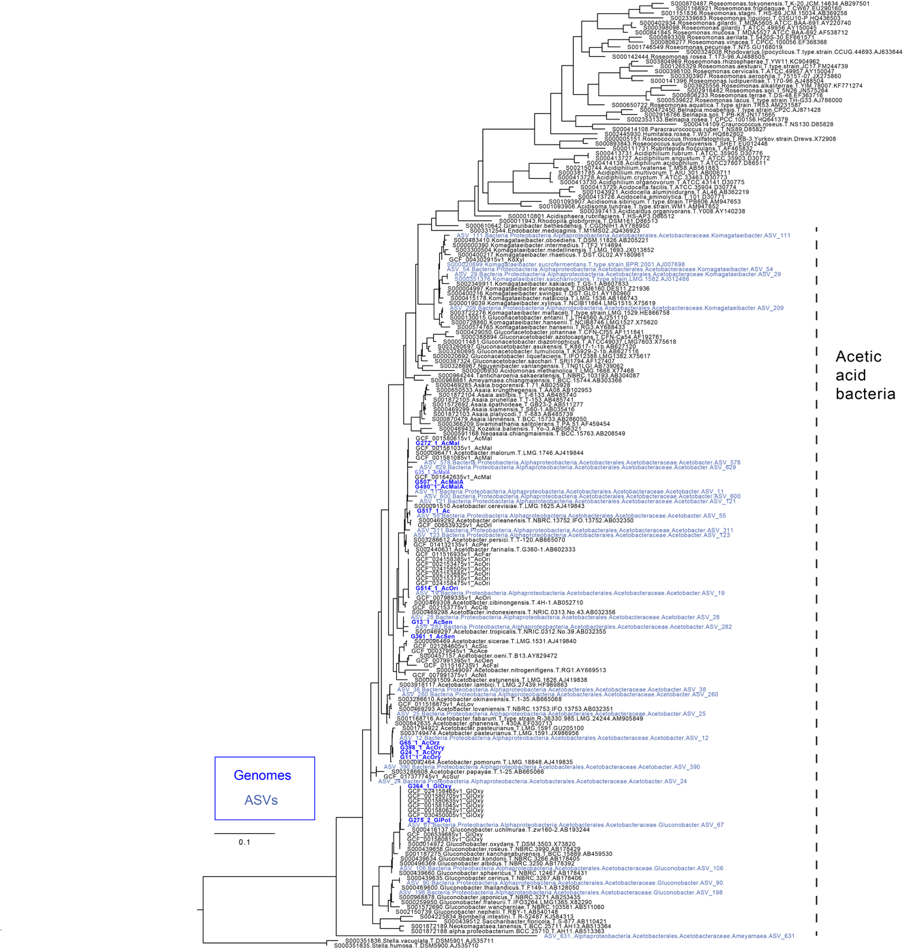
**

**Fig. S1: Phylogenetic tree of 16S rRNA gene sequences (N = 197) to determine ASV taxonomic assignments.** When present, full or partial 16S rRNA genes were extracted from isolate genomes, MAGs, and NCBI genomes. Only AAB recovered are shown here, along with an outgroup. A phylogenetic tree was built using RAxML, and ASV taxonomic assignments were then made to the nearest genome representative (see Methods for additional details).

**Fig. S2: Full relative abundances of AAB ASVs in 500 sourdough starters.** Percent relative abundance of AAB by species taxonomic assignments, summarized in Fig. 2A. Some ASVs could only be assigned to a nearest species cluster.

**
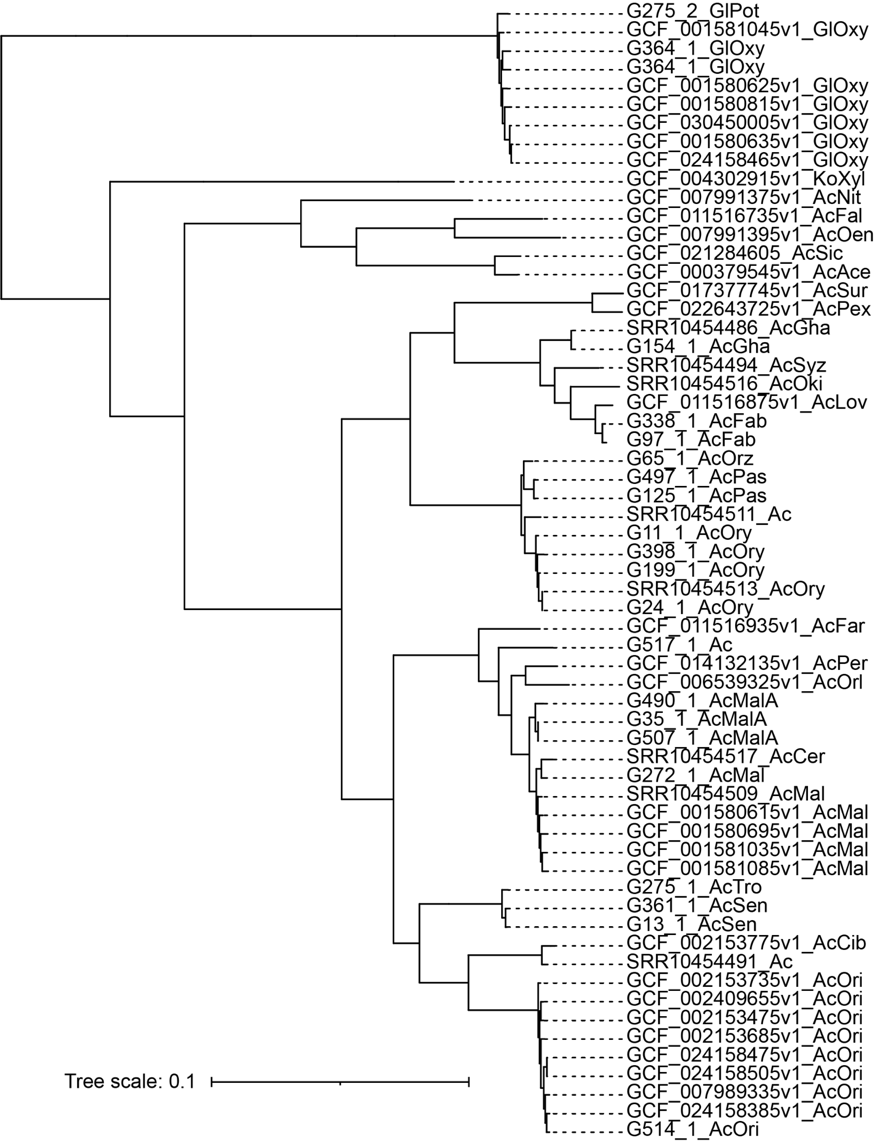
**

**Fig. S3: Phylogenetic tree of 61 AAB genomes included in analyses.** Genome tree built with KBase SpeciesTree v2.2.0 from AAB genomes isolated from sourdough, assembled from sourdough metagenomes, and downloaded from NCBI.

**
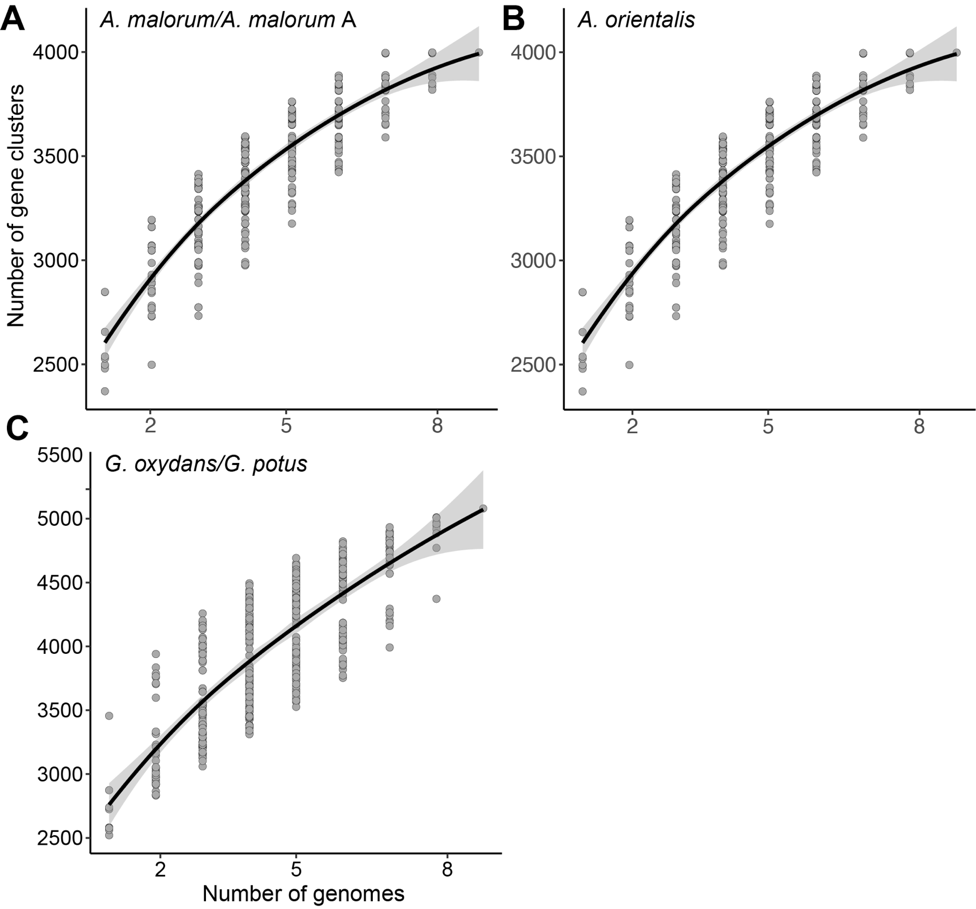
**

**Fig. S4: Pangenome plots: number of genomes by shared gene content.** Accumulation plots visualizing number of genomes included in pangenome and the resulting number of unique gene clusters for **(A)** *A. malorum/A. malorum A*, **(B)** *A. orientalis* and **(C)** *G. oxydans/G. potus.* Each point represents a unique set of genomes, and at every distinct number of genomes, all possible combinations of genomes were subsampled out of the total (N = 9 for each). Curves show smoothed conditional means (loess) and gray shade indicates standard error.

**
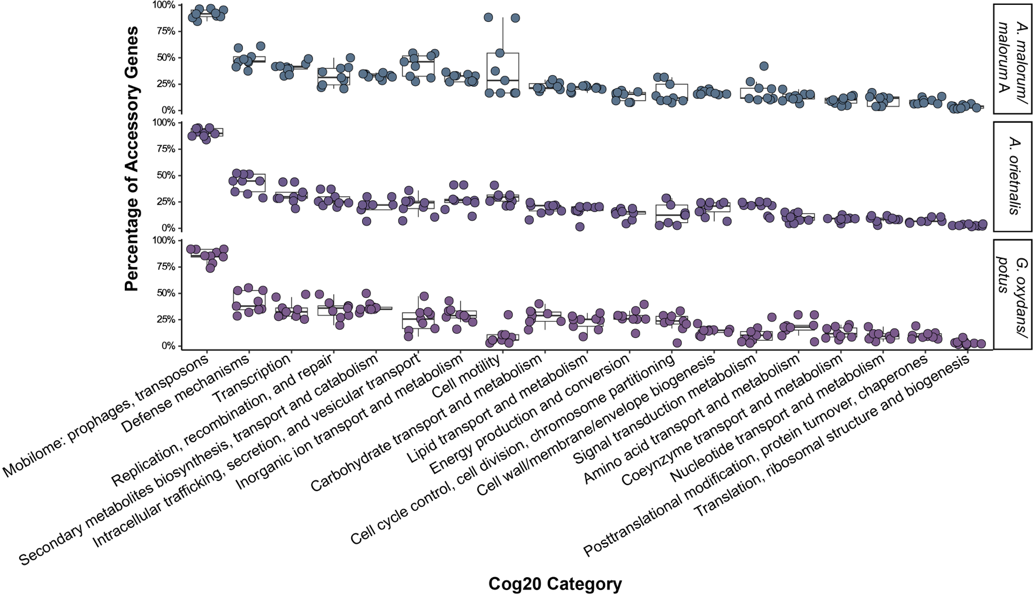
**

**Fig. S5: Full summary of core vs. accessory COG categories.** Percentage of genes in the accessory genome within all functional categories of COG annotations across the three species highlighting strain diversity (*A. malorum*/*malorum* A, *A. orientalis*, and *G. oxydans*/*potus*).

**
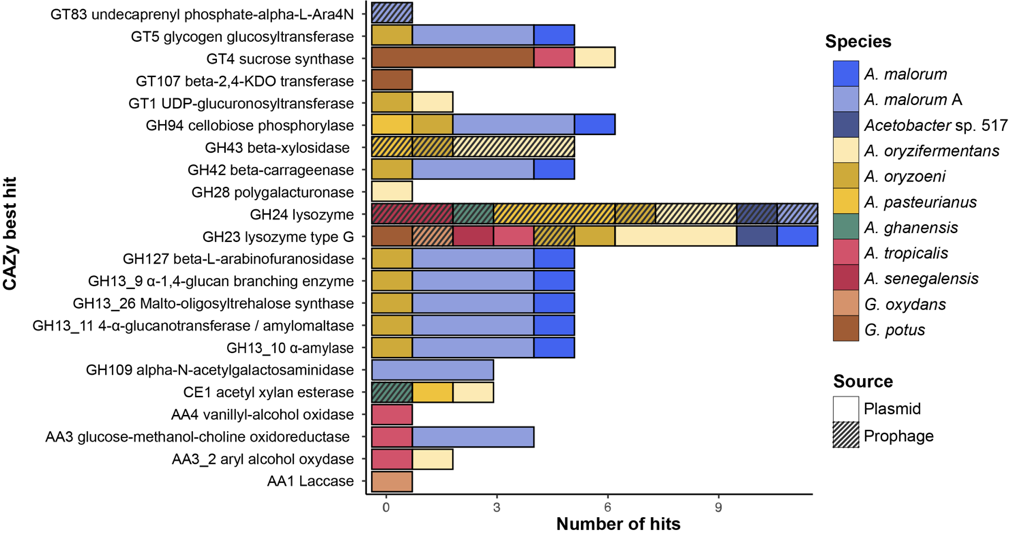
**

**Fig. S6: CAZymes are carried on mobile elements in AAB genomes from sourdough.** Number of hits to CAZyme genes detected within sourdough AAB genomes, colored by species, and patterned by source, either on plasmids or integrated from prophages.

**
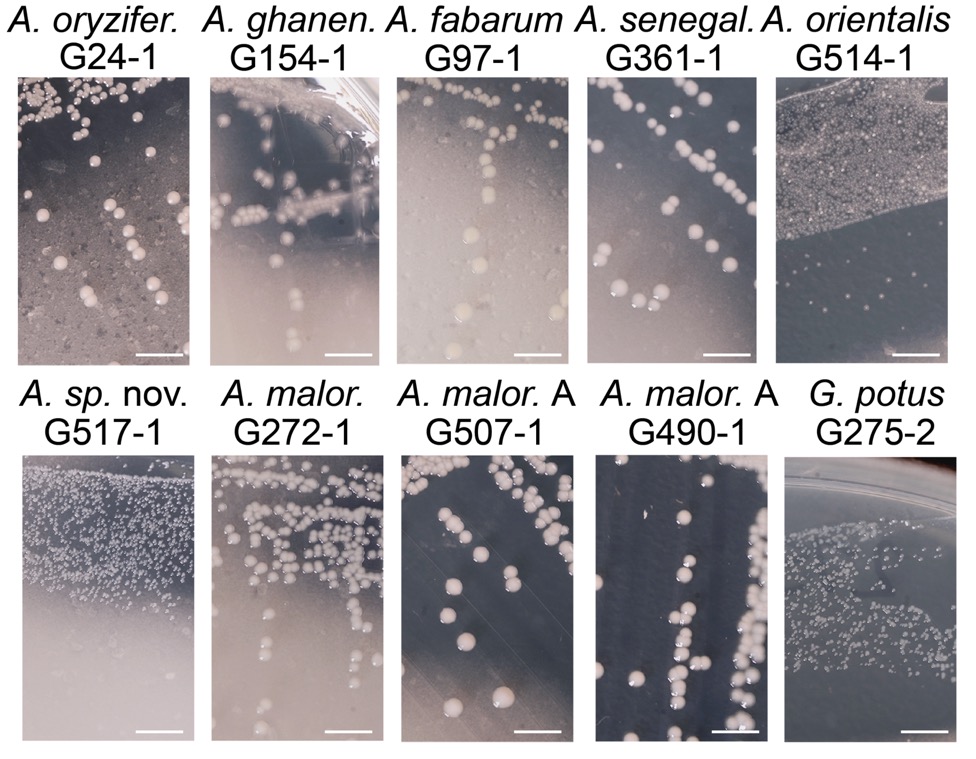
**

**Fig. S7: Variation in colony morphology of sourdough AAB.** Ten strains of acetic acid bacteria selected for synthetic starter experiments plated on GYCA with calcium carbonate, imaged with a Sony Alpha 7RIII. Scale bars represent approximately 2mm.


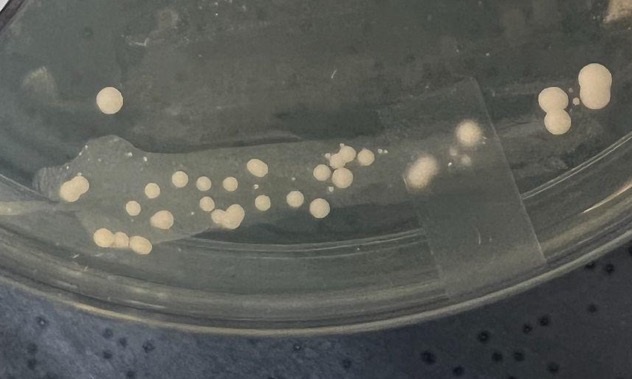


**Fig. S8: Yeast appear glued in exopolysaccharides from *A. orientalis*.** SynCom361 community A3 (*S. cerevisiae, L. brevis, A. orientalis*) well 8 streaked for presence/absence on day four onto Yeast Potato Dextrose plate with chloramphenicol (selective for yeast). **
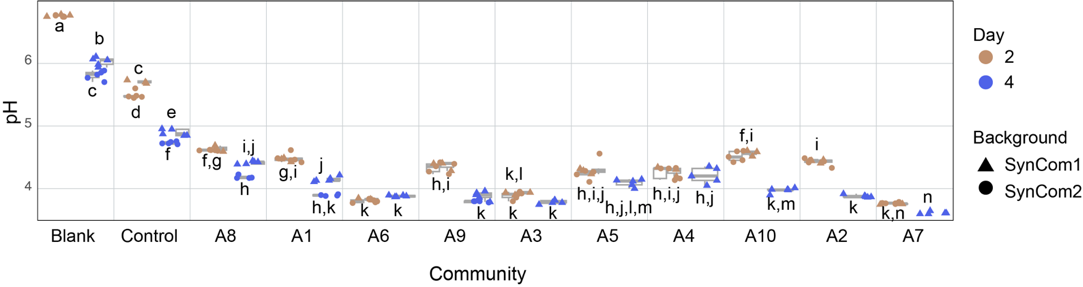
**

**Fig. S9: Diverse AAB species and strains all acidify the sourdough starter environment relative to the yeast and LAB-only controls and strain level differences in emergent acidification are highly conserved.** To assess acidification of the overall microbiome with and without AAB strains, we measured the pH at day 2 (after 48 hours) and day 4 (after 96 hours) of five replicate consortia for every distinct treatment. There were 204 distinct pH measurements, representing ten AAB treatments, a blank, and a YL control, with five replicates each across the two timepoints. We excluded two mis-transferred wells and a subset of treatments from SynCom361 day 4 (N = 34) where yeast failed to persist. Different letters represent post hoc significant pairwise differences.
