# Supplementary material for: Genomics and synthetic community experiments uncover the key metabolic roles of acetic acid bacteria in sourdough starter microbiomes
Source: mSystems. 2024 Sep 17;9(10):e00537-24. doi: 10.1128/msystems.00537-24 (PMC11498085; doi:10.1128/msystems.00537-24)
Supplement: Captions — for Tables S1 to S14. [file msystems.00537-24-s0002.docx]

**Supplemental table legends:**

**Table S1.** Species’ assignments of AAB ASVs recovered from 500 sourdough starters.

**Table S2.** Yeast and LAB ASVs statistically enriched in AAB-dominant sourdough starters.

**Table S3.** Co-occurrences of ASVs with AAB in sourdough starters.

**Table S4.** Metadata of 61 AAB genomes, including species, sources, genome stats, and genome accessions.

**Table S5.** ANI matrix of 61 AAB genomes by percent ANI between all genomes.

**Table S6.** Number of gene clusters recovered in AAB pangenomes with core and accessory distributions.

**Table S7.** Summary of microbial metabolic traits across 61 AAB genomes output from DRAM.

**Table S8.** Functional enrichments by environment (sourdough or other) across 61 AAB genomes (Anvi’o).

**Table S9.** Functional enrichments by environments (sourdough or other; Kruskal-Wallis) between AAB strains.

**Table S10.** Annotation outputs of sourdough AAB genes recovered on plasmids or from prophages.

**Table S11.** Analyses by plate and well of synthetic starter communities, including presence/absence, pH, CFU counts, and VOCs.

**Table S12.** Volatile organic compound peak areas of synthetic sourdough communities. Similarity scores match with reference ions, CAS = Chemical Abstract Service, Rt = Retention time, QC = quality control samples.

**Table S13.** VOC means and statistical differences in compound relative abundance between sample clusters.

**Table S14.** Statistical outputs relating differences in function (including pH and VOC profiles) and composition (AAB CFUs) to differences in treatments (including treatment type and differences in ANI across AAB strains).
